# Standardised tobacco packaging: a health policy case study of corporate conflict expansion and adaptation

**DOI:** 10.1136/bmjopen-2016-012634

**Published:** 2016-10-07

**Authors:** Jenny L Hatchard, Gary J Fooks, Anna B Gilmore

**Affiliations:** 1 Tobacco Control Research Group, Department for Health, University of Bath, Bath, UK; 2 School of Languages and Social Sciences, Aston University, Birmingham, UK

**Keywords:** Tobacco, Corporations, Policy, Transparency, Packaging

## Abstract

**Objectives:**

To investigate opposition to standardised tobacco packaging in the UK. To increase understanding of how transnational corporations are adapting to changes in their access to policymakers precipitated by Article 5.3 of the Framework Convention on Tobacco Control (FCTC).

**Design:**

Case study web-based documentary analysis, using NVivo V.10. Examination of relationships between opponents of standardised packaging and transnational tobacco companies (TTCs) and of the volume, nature, transparency and timing of their activities.

**Setting:**

UK standardised packaging policy debate 2011–2013.

**Participants:**

Organisations selected on basis of opposition to, or facilitation thereof, standardised tobacco packaging in the UK; 422 associated documents.

**Results:**

Excluding tobacco manufacturing and packaging companies (n=12), 109 organisations were involved in opposing standardised packaging, 82 (75%) of which had a financial relationship with 1 or more TTC. These 82 organisations (43 actively opposing the measure, 39 facilitating opposition) were responsible for 60% of the 404 activities identified, including the majority of public communications and research production. TTCs were directly responsible for 28% of total activities, predominantly direct lobbying, but also financially underwrote third party research, communication, mass recruitment and lobbying. Active organisations rarely reported any financial relationship with TTCs when undertaking opposition activities.

**Conclusions:**

The multifaceted opposition to standardised packaging was primarily undertaken by third parties with financial relationships with major tobacco manufacturers. Low levels of transparency regarding these links created a misleading impression of diverse and widespread opposition. Countries should strengthen implementation of Article 5.3 of the FCTC by systematically requiring conflict of interest declarations from all organisations participating in political or media debates on tobacco control.

**Strengths and limitations of this study**

- This paper is the first study to systematically examine the scale and nature of opposition to standardised tobacco packaging outside Australia.
- While tobacco industry document research has been used extensively to examine the *historical* political activity of corporations, this study employs an innovative combination of publicly available sources and investigative research techniques to analyse corporate political action in *contemporary* health policy conflicts.
- The study presents a novel classification system for systematically examining the sector and relationships of ostensibly distinct organisations opposing public health policy which can be applied in other settings.
- The ongoing nature of the conflict during the study period restricted the study to examining political activity, rather than influence.
- Further research using social network and social media analysis would provide deeper insights into how protobacco policy networks are formed and operate.

## Introduction

On National No Smoking Day, 11 March 2015, over 6 years after it was first proposed^1^ and following its inclusion in numerous policy initiatives,^2–4^ the UK House of Commons voted to introduce standardised packaging for tobacco products (see online supplementary file 1).^5^ ^6^ In jurisdictions with advertising^7–9^ and point of sale display restrictions,^10–13^ packaging is (alongside product design) one of transnational tobacco companies' (TTCs) few remaining forms of brand marketing and identity.^14–17^ By prohibiting logos, brand imagery and promotional text, standardised packaging aims to reduce the appeal of tobacco products and increase the effectiveness of health warnings.^18–25^ Academic and government reviews of the evidence have concluded that the policy is likely to reduce youth smoking uptake.^18^ ^26–34^

10.1136/bmjopen-2016-012634.supp1 supplementary data

Standardised packaging is the second major policy restricting tobacco companies' commercial activities (the first being the Point of Sale Display Ban) to be proposed in the UK following the introduction of both restrictions on tobacco industry political activity under Article 5.3 of the Framework Convention on Tobacco Control (FCTC)^2^ ^35^ ^36^ and policy instruments associated with the Better Regulation agenda.^37–40^

Article 5.3 of the FCTC requires governments to ‘protect’ tobacco control policies ‘from commercial and other vested interests of the tobacco industry’.^35^ ^36^ In order to meet this requirement, the UK Government committed to placing significant restrictions on tobacco companies' capacity to use ‘insider’ political strategies, such as direct lobbying, to influence tobacco policy.^41^ ^42^ Insider strategies are characterised by a close relationship with policy élites and are based on exchange of information and mutual trust and support.^42^ In response to Article 5.3, the government undertook to publish the details of all policy-related meetings between the tobacco industry and government departments and to require organisations engaging with the Department of Health on tobacco control to disclose any relevant relationship with the industry.^3^ These changes work to formalise tobacco manufacturers' status as political ‘outsiders’.^4^ ^5^

In contrast, reforms under the Better Regulation agenda require that all new policy proposals undergo both stakeholder consultation and impact assessment, for which submissions and data from industry are specifically invited.^43–46^ This creates a new, highly accessible, evidence-focused policymaking venue, into which business and civil society actors can feed information.^40^ ^43^ ^47^ ^48^

This study is the first to explore how major tobacco manufacturers are negotiating this new institutional context in the UK. It is also the first European study to examine their political action in response to standardised tobacco packaging proposals, a policy which was first implemented in Australia in 2012 and is being taken up by other jurisdictions (eg, Belgium, Bulgaria, Finland, France, Hungary, Ireland, Norway, Sweden, Canada and New Zealand). Using an innovative approach to tobacco industry research, the study analyses publicly available sources to explore contemporary, rather than historical,^49^ political activity. It aims to identify *relationships* between TTCs and those opposing standardised packaging and to examine the *volume* and *nature* (type and range) of activities undertaken by them to oppose the policy in the UK between 2011 and 2013. It further explores the *transparency* of opposition organisations' relationships with TTCs and assesses the *timing* of their activities vis-à-vis key events in the policy process.

## Methods

### Data collection and recording

In 2012, the Department of Health held a 4-month consultation (April to August) on standardised packaging for tobacco products. This attracted the largest ever response to a public consultation in the UK, and featured both strong opposition to and significant support for the measure (see online supplementary file 1).^3^ The study mapped opposition to standardised packaging during the 3-year period (1 January 2011–31 December 2013) straddling the consultation.^50^ ^51^ Data collection began in January 2013 and was prospective and retrospective. Using snowball sampling, data were predominantly collected from online sources, accessed via search engines and organisational website search functions. To facilitate this approach, web monitoring was employed, using both ‘ASH Daily News’ and daily search engine alerts for key words: *packaging, plain, tobacco, cigarette, UK*. Data were also gathered from freedom of information (FOI) requests made to the Department of Health, Intellectual Property Office and the Treasury. These requests asked for ‘meetings, meeting notes, agendas, records of telephone conversations, or email or written correspondence with or from tobacco companies, lobby groups (eg, retail, business, trademarks) and/or think tanks where standardised or plain packaging of tobacco products was mentioned/discussed’ specifying dates relevant to the study timeframe. Semistructured interviews (between 45 and 60 min) were conducted with seven public health advocates to obtain background information and source further data. The study was also informed by leaked documents from Philip Morris International (PMI).^52^ ^53^ Written consent was provided by all interviewees.

Four hundred and twenty-two data items (including letters, videos, web articles and news items, press releases, adverts, reports) were identified. Data were imported into NVivo V.10 and recorded in a classification spreadsheet. The sample excluded blog posts, social media entries, correspondence sent in response to requests or enquiries from government departments, and consultation submissions (including associated cover letters), the latter having been analysed at length in other studies.^54–56^

### Data coding

Data were coded for actor characteristics and political activity themes (table 1). In terms of inclusion criteria, ‘actors’ were defined as companies, organisations and groups whom the data showed undertook political activity to oppose, or to facilitate opposition to, standardised packaging. They were classified according to their *role* (identified from data), *sector* (identified from data and actors' websites) and *relationship with major tobacco companies*. Relationship was identified using a four step process: (1) data; (2) actors' websites, transparency registers and databases; (3) general web searches combining organisation names with TTC names; and (4) email enquiries.^54^ Political activities were coded deductively using themes based on Savell *et al*.^57^ The transparency of TTC involvement in activities was also coded from the data (table 1). All data were coded by JLH and discussed and agreed iteratively with GJF. Triangulation, prolonged engagement with the context, persistent observation of the data and peer debriefing were employed to ensure the validity of findings.^59^

**Table 1:** Classification system for participants and their activity in the conflict

*Classification of actors*	
Role—determined from data	
Active participants	Actors who opposed standardised packaging in public, business and/or political venues
Facilitative participants	Actors who did not oppose standardise packaging on their own behalf, but undertook research or other consultative work for active participants that was subsequently used by the active participants, for example, to develop and substantiate arguments and disseminate them for public and political audiences
Sector—determined from data and actors' websites	
Academia	Universities
Business	Tobacco product and tobacco packaging manufacturing and tobacco packaging design companies Non-manufacturing companies General and sectoral business associations
Civil society	Think tanks General and smokers’ rights groups Labour unions Retired police associations
State	Parliamentary groups
Relationship with tobacco companies—determined from data, actors’ websites, transparency registers, general internet searches and email enquiries^54^	
Financial	Core funding from one or more TTC Campaign funding from one or more TTC Membership funding or donation from one or more TTC Client relationships with one or more TTC
Non-financial	Employee membership (where TTC employees were members of organisations) Third party connections (where an indirect link exists between the actor and a TTC via a third party)
None	No relationship between the actor and any of the four major tobacco companies
Unknown	Insufficient information to determine whether a relationship existed
Manufacturers	Tobacco product and packaging manufacturing companies were exempt from classification for relationship
*Classification of political activity*	
Type of political activity	
Research production	The commissioning and production of policy-relevant research
Public communication	Public communication of arguments to the general public and to sectoral audiences (eg, retailers, smokers) via the mainstream and sectoral media, including press, online, films, events
Mass recruitment	Mass recruitment of the general public and of particular sectors to encourage responses to the 2012 consultation and communication with MPs and Ministers
Direct lobbying	Direct lobbying of politicians and civil servants via hospitality, meetings, events and publications and correspondence
Transparency of involvement of TTCs in political activity	
Explicit	Clear declaration of TTCs funding or involvement in activity-related documents
Implicit	Activity-related documents did not include a declaration of TTC funding, but one could be found on the publishing website
Undeclared	Neither documents nor publishing website included a declaration of TTC funding
Not applicable	No evidence of TTC involvement in political activity

MPs, Members of Parliament; TTC, transnational tobacco company.

**Table 2:** Number, sector, role and relationships with TTCs of organisations (n=121) opposing standardised packaging of tobacco products UK 2011–2013

Category	Sector	Total number of (%) organisations per category, n=121	Roles		Relationships with TTCs, n=109*			Political activity, n=404			
			Number of active organisations	Number of facilitative organisations	Number of (%) financial relationships with tobacco companies^58^	Number of (%) non-financial relationships with tobacco companies	Number of (%) no relationship with tobacco companies identified	Research	Public communication	Mass recruitment	Direct lobbying
Business (tobacco)	Tobacco manufacturers	12 (10)	4	0	NA	NA	NA	1/C	22/C	1/C	90/C
	Packaging and design companies		8	0	NA	NA	NA	–	–	4	7
Business (other)	Investment banks	35 (29)	1	0	0 (0)	1 (100)	0	1	–	–	–
	Media companies		2	0	1 (50)	1 (50)	0	–	3	1	2
	Law firms†		1	5	5 (83)	1 (17)	0	4	2	–	5
	Public relations companies†		1	6	7 (100)	0	0	–	2/C	C	3
	Research consultancies†		1	18	17 (89)	2 (11)	0	31	4	–	–
Business associations	Manufacturing	35 (29)	2	0	2 (100)	0	0	–	5	–	3
	Packaging		1	0	0	0	1 (100)	–	–	1	–
	Wholesale		2	0	2 (100)	0	0	–	7	1	2
	Retail		10	0	8 (80)	0	2 (20)	–/C	45	4	7
	General		9	0	9 (100)	0	0	–	1	–	4
	Intellectual property		11	0	8 (73)	1 (18)	2 (9)	–/C	9	–	7
Civil society	Think tanks	24 (20)	13	0	6 (46)	7 (54)	0	7	32	–/S	1
	General rights groups		5	0	2 (40)	2 (40)	1 (20)	–	22	–	1
	Smokers’ rights groups		2	0	2 (100)	0	0	–	38	1	5
	Unions representing tobacco employees		2	0	0	2 (100)	0	–	–	1	–
	Retired police groups		2	0	0	1 (50)	1 (50)	–/C	1	–	–
Academia	Universities†	14 (11.6)	0	14	13 (93)	1 (7)	0	13	–	–	–
State	Ad hoc parliamentary alliances	1 (<1)	1	0	0	1 (100)	0	–	–	–	3
Total		121	78	43	82 (75.2)	20 (18.4)	7 (6.4)	57	193	14	140

Political activity: sectors which did not undertake specific political activities.

*Excludes tobacco and packaging companies.

†Organisations commissioned to provide research, legal and public relations services to tobacco companies and other active organisations in the conflict.

C, commissioned or collaborated in activity; NA, not available; S, supported mass recruitment activities but did not initiate them; TTC, transnational tobacco company.

### Analysis

First, we examined the data for number of organisations involved, their role in the conflict, their sector and their relationship with TTCs. Second, the volume, type, nature, timing and transparency of the different political activities undertaken by each actor were systematically quantified. This information was used to understand the pattern of activity by sector and relationship with TTCs. Third, interpretive qualitative analysis was used to obtain a deeper understanding of the purpose of the different types of activities identified.

## Results

### Organisations opposing standardised packaging and their relationships with TTCs

One hundred and twenty-one organisations were identified as opposing standardised packaging within the study period (table 2). Of these, 78/121 (64.5%) actively opposed the policy (active participants). In total, 43/121 (35.5%) were facilitative participants who provided legal, research and public relations services to active organisations, without directly opposing the policy on their own account.

**Figure 1:**
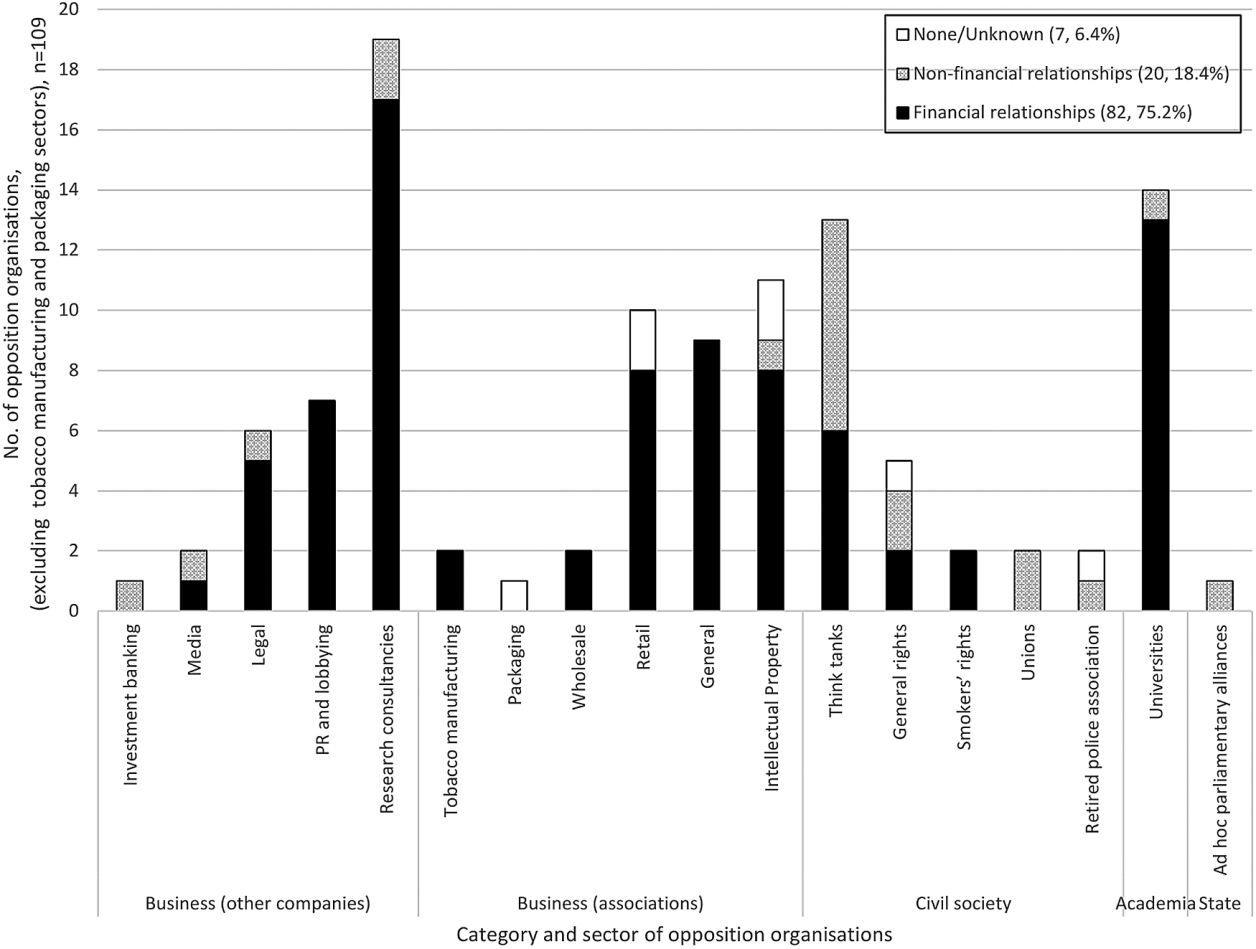
Number, sector and relationship with TTCs of organisations opposing, or facilitating opposition to, standardised packaging in the UK 2011–2013 (excludes tobacco manufacturing, packaging and design companies), n=109. PR, public relations; TTC, transnational tobacco company.

Only 12/121 (10%) were businesses from the tobacco or tobacco packaging manufacturing sectors (table 2). The remaining 109 organisations comprised: 35/121 (29%) non-manufacturing businesses; 35/121 (29%) business associations; 24/121 (20%) civil society organisations; 14/121 (11.6%) universities and 1/121 (<1%), an ad hoc parliamentary alliance (table 2 and figure 1).

Of these 109 organisations, 82/109 (75.2%) had financial relationships^58^ and 20/109 (18.4%) non-financial relationships with at least one TTC (table 2 and figure 1). The remaining 7/109 (6.4%) organisations comprised one which was found to have no connection with any tobacco company, and six which could not be classified due to insufficient information.

Ten of the organisations with financial relationships with TTCs were found to be in receipt of core and/or antistandardised packaging campaign funding, 30 had tobacco company members or had received tobacco company donations, and 42 had tobacco company clients. One hundred per cent of smokers' rights groups, public relations and lobbying firms, manufacturing, wholesale and general business associations were found to have financial relationships with TTCs (table 2). Ninety per cent or more of universities, and 80% or more of law firms, research consultancies and retail associations identified in the research were also financially linked to TTCs. Of the 20 organisations with non-financial relationships, 17 were connected with tobacco companies via a third party. For example, organisations were linked to TTCs via bridging actors engaged in either neoliberal or business networks *and* tobacco company-funded activity. Others were subcontracted by organisations commissioned by tobacco companies or had collaborated with tobacco company consultants.^54^ Three included tobacco company employees among their members.

**Table 3:** Examples of ‘insider’ and ‘outsider’ activities undertaken by TTCs and opposition organisations

Political strategy type	Political activities*	Extracts
Insider strategy: research production and direct lobbying	PMI commission an opinion from Lord Hoffman, which they and public relations firm, Crosby Textor Fullbrook (retained by PMI), promote to ministers and officials in the Intellectual Property Office.^60^	*“A prohibition on the use of a mark is in my view a complete deprivation of the property in that mark, notwithstanding that the proprietor might be able to distinguish his goods by the use of some other mark.”* Lord Hoffman, *Philip Morris International: Opinion*, 24.05.12^61^ “*I will send you the Lord Hoffman opinion in the near future as I'm sure it would be of interest to you.”* Philip Morris Ltd email to the Intellectual Property Office, *Fw: Meeting follow up—21 June 2012—Email 1* 25.06.12^62^ “*My dear Lord, please find enclosed as promised a telling opinion from Lord Hoffman…Hoffman is the most telling and concerning from an IP viewpoint.”* Lynton Crosby, Crosby-Textor-Fullbrook, email to Lord Marland, Minister for Intellectual Property, 01.11.12^63^
Outsider strategy: public communication and mass recruitment	BAT, ITG and JTI core-fund active organisations (Forest, Hands Off Our Packs (HOOPs)) who mobilise support from other organisations to help promote their antistandardised packaging messaging, eventually generating nearly 270 000 antistandardised packaging signatures.^3^ ^64^	*“Angela Harbutt [head of HOOPs]… believed the proposal was the most ill-conceived, idiotic and illiberal idea that had come out of any UK government during the past five or 10 years. She said that it was a fight that had to be won, and she asked those present to sign up to the campaign and to spread the word.”* Forest on the HOOPs campaign launch, 27.02.12^65^ “*When the government came to power, I don't remember them saying it was going to be a priority for them to increase regulation and persecute minority lifestyle choices. But that's what this consultation is about….So please sign up to the HOOPs campaign and do everything you can to stop this ridiculous new growth of the nanny state.”* Mark Littlewood, Director, Institute of Economic Affairs, in HOOPs’ campaign film, *Nannytown*, 07.03.12^66^ “*It's about time we called a halt to the advance of the ‘nanny state’ and reasserted the historic British values of tolerance and freedom.”* Andrew Turner, CEO, API Group (packaging), interviewed by HOOPs, 01.05.12^67^

*For more information on these cases see http://www.tobaccotactics.org.

PMI, Philip Morris International; TTC, transnational tobacco company.

### Opposition organisations' political activity

Political activities were extensive (n=404, identified from the 422 documents) and entailed four main types of action: production of policy-relevant research (57, 14%); public communication (193, 48%); mass recruitment (14, 3%) and direct lobbying (140, 35%).

**Figure 2:**
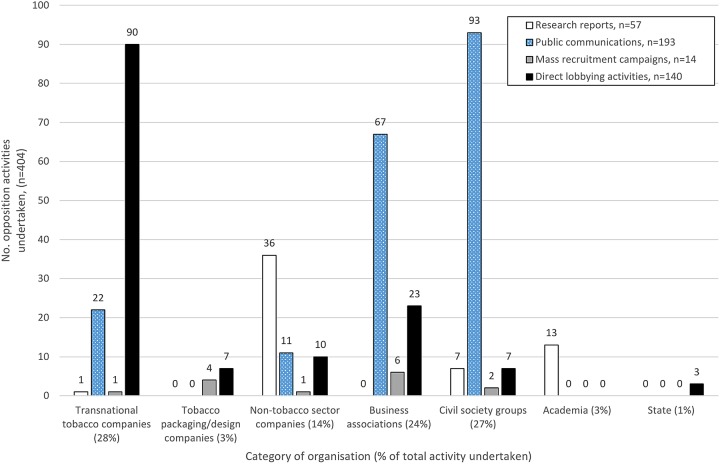
Volume and distribution by sector of activities undertaken or cited to oppose standardised packaging 2011–13, n=404.

Different sectors focused on different types of activity (table 2 and figure 2). TTCs (114/404, 28%) were particularly active in direct lobbying of government. Civil society organisations (109/404, 27%) and business associations (96/404, 24%) prioritised public communications. Other companies (58/404, 14%), predominantly comprising law and public relations firms, and academic actors (13/404, 3%) mainly produced research. Packaging and design firms undertook a small number of mass recruitment and direct lobbying activities (11/404, 3%). State actors only undertook direct lobbying (3/404, 1%).

**Figure 3:**
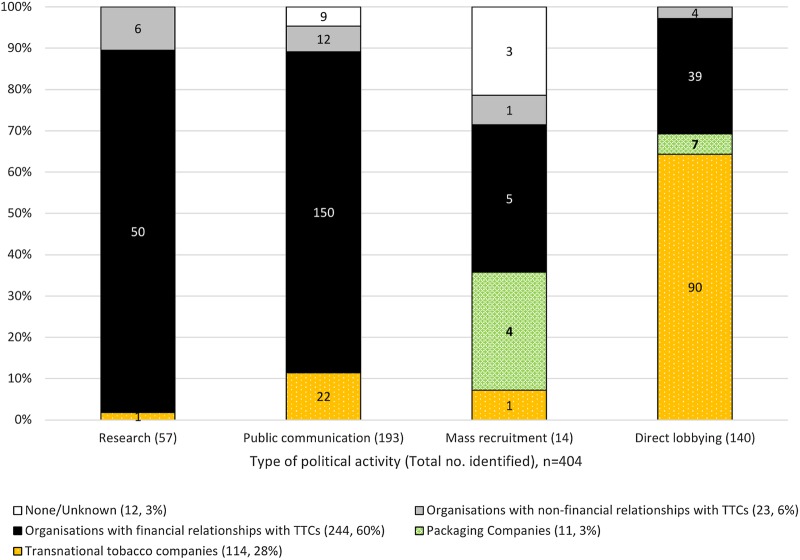
Distribution of types of political activity by relationship of organisations with TTCs, n=404. TTC, transnational tobacco company.

The distribution of opposition activities also varied with organisations' *relationships* with TTCs (figure 3). Organisations financially related to TTCs undertook the majority (244/404, 60%) of all opposition activities, including nearly 9 out of 10 research reports and three-quarters of public communications. Organisations with non-financial relationships undertook all four activity types (23/404, 6%; figure 3). The data reveal how activities combined to form both ‘insider’ and ‘outsider’ political strategies. For example, research produced by third parties was frequently cited in lobbying activities and business and civil society organisations were found to collaborate on public communications and mass recruitment campaigns (table 3).

#### Production of research

Nearly 9 of every 10 research reports (50/57, 88%) were authored by organisations with a financial relationship with TTCs (figure 3). They presented similar arguments to tobacco company consultation submissions, where they were widely quoted,^55^ and to PMI's antistandardised packaging strategy (leaked to the public health non-governmental organisation, Action on Smoking and Health).^52^ ^53^ These were: negative economic, illicit trade, intellectual property and trade and price consequences of standardised packaging; criticism of the policy process; and lack of evidence of effect. The data showed that commissioned research reports were used by TTCs and financially linked actors to support opposition arguments in public communication and direct lobbying activities.

#### Public communications

Over three-quarters (150/193, 78%) of public communications were undertaken by 35 business associations, civil society groups and a public relations firm with financial relationships with TTCs (figure 3). Public communications replicated TTCs' main arguments against standardised packaging, and disseminated them to general and sectoral audiences. General audience communications included press releases, films raising the spectres of illicit tobacco and the ‘nanny state’,^66^ ^68–72^ advertisements subsequently judged to be in breach of the Advertising Standards Authority's code^73–77^ and articles in the mainstream media. Sectoral communications included articles, news stories and events directed at specific groups, such as retailers and intellectual property professionals.^78–81^ The combination of general and sectoral communications found in the data suggests that TTCs, and other opponents of standardised packaging, were cognisant of the power of different media to raise the significance of this policy among different constituencies. Such communications may have contributed to the intensity of the 2012 consultation response (see *mass recruitment*) and indirectly to the period of seeming political inactivity which followed.

#### Mass recruitment

Five mass recruitment campaigns were undertaken by organisations financially linked to TTCs and one by Imperial Tobacco Group themselves (figure 3 and table 4). Campaigns aimed to encourage the general public and targeted constituencies to submit postcard, standard letter or petition-style responses to the 2012 consultation and to lobby Members of Parliament (MPs) and Ministers.^3^ ^52^ ^53^ Campaign materials were promoted online, in the workplace and in the street and reflected TTC arguments.^83^ ^85^ ^86^ The largest campaign, Hands Off Our Packs, extended its reach by commissioning street marketing in 30 UK cities^87^ and collaborating with sympathetic organisations (table 3).^66^ ^88–92^ The five mass recruitment campaigns directly funded by TTCs generated 420 394/427 812 (98%) opposition campaign submissions.^3^ Mass recruitment campaigns aimed to translate opposition-oriented general and sectoral opinion (underpinned by public communications) into mass political action.

**Table 4:** Mass recruitment campaigns to oppose standardised tobacco packaging, n=14

Actor information				Mass recruitment campaign details			
Category	Sector	Name	Relationship	Name of campaign	Tobacco company funding?	Recruitment targets	Number of submissions
Business (tobacco)	Tobacco manufacturers	Imperial Tobacco	Direct commercial	Say NO to plain packs	ITG	General public	120 247
	Packaging and design companies	Benkert	Direct commercial	(Unnamed)	NA	Packaging employees	131
		Chesapeake	Direct commercial	Say ‘No’ to standardised packaging	NA	Packaging employees	79
		Parkside Flexibles	Direct commercial	(Unnamed)	NA	Packaging employees	196
		Weidenhammer	Direct commercial	(Unnamed)	NA	Packaging employees	869
Business (other)	Media companies	Asian Media and Marketing Group	Financial	(Unnamed)	PMI, ITG^82^	Retailers	898
Business associations	Packaging	Unnamed packaging employees group	Unknown	(Unnamed)	NA	Packaging employees	175
	Wholesale	Scottish Wholesale Association	Financial	Plain Nonsense	BAT, ITG^83^ ^84^	Unknown	2865
	Retail	Association of Independent Tobacconists	Unknown	(Unnamed)	NA	Specialist tobacconists and customers	3199
		National Federation of Retail Newsagents	Financial	(Unnamed)	NA	Retailers	6
		Tobacco Retailers’ Alliance	Financial	No to ‘plain’ packs	BAT, ITG, JTI	Retailers	26 530
		Unnamed retailers’ group	Unknown	(Unnamed)	NA	Small retailers	561
Civil Society	Smokers’ rights	Hands Off Our Packs and Forest	Financial	Hands Off Our Packs	BAT, ITG, JTI	General Public	269 854
	Unions	Unite and GMB	Non-financial	Plain packaging of tobacco products: Caution UK jobs at risk	NA	Tobacco, print and packaging employees	2202
Total							427 812

Source: Department for Health.^3^ The figure 427 812 is at variance with that quoted in the Department of Health report (427 888) as it excludes 2 letters co-signed by 51 MPs and 25 former policemen. These are included elsewhere in the analysis of political activity in the paper.

MPs, Members of Parliament; NA, not available.

#### Direct lobbying of policymakers

Over a quarter (39/140, 28%) of direct lobbying was undertaken by organisations financially linked to TTCs and nearly two-thirds (90/140, 64%) by TTCs themselves (figure 3). Direct lobbying included: 32 hospitality gifts valued at £32 583 from JTI;^93^ 17 meetings with government officials at the Treasury, Department of Health and Intellectual Property Office;^62^ ^94–101^ 9 events, including in Parliament on the illicit trade^102–106^ and party conferences;^107^ ^108^ publications in parliamentary magazines;^82^ ^84^ ^109^ and 82 correspondence items sent mainly to the Department of Health, the Treasury and the Intellectual Property Office.^110–120^ Correspondence included letters from an ad hoc group of 50–74 MPs, 8 of whom had previously accepted gifts from JTI.^93^ ^121–123^ Direct lobbying activities drew on the range of arguments promoted by TTCs, and flagged or enclosed industry-commissioned research reports (table 3).

#### Transparency of tobacco industry involvement in opposition activities

*Active* organisations with financial relationships with TTCs (n=43) were transparent in only half of correspondence (15/31, 48%), 1 of 4 research reports^124^ and <1 in 5 public communications (27/150, 18%). For example, in public communications former police officers did not declare membership of the Common Sense Alliance (a FOREST offshoot).^125–129^

In contrast, TTCs (n=4) and *facilitative* actors with financial relationships with them (n=39) were transparent in reporting their interests. All but one research reports (41/42, 98%) declared their source of funding, although references to authorial independence, experience and qualifications were used to offset potential negative effects of corporate funding on credibility.^55^ Yet, when promoted by *active* participants in the conflict, TTC funding of research was only acknowledged in 6/20 (30%) citations in press releases^130–135^ and in 4/35 (11%) citations in direct lobbying correspondence.^136–139^

#### Timing of political activity

**Figure 4:**
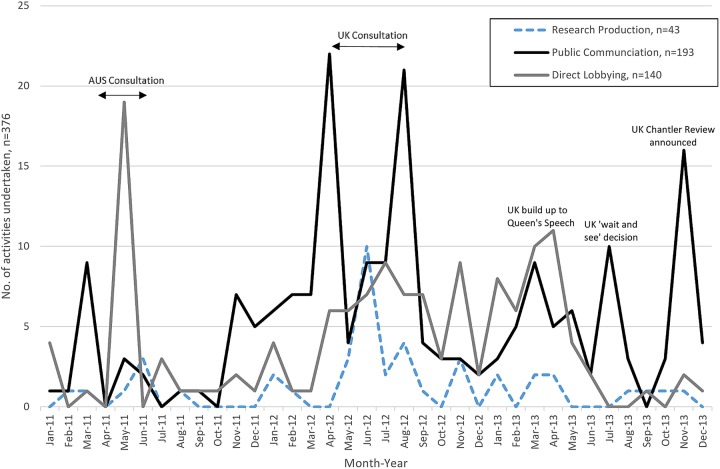
Timing of opposition political activity 2011–2013, n=376 (excludes 14 mass recruitment campaigns which occurred over a series of months and 14 research reports which were published between 2008 and 2010 but were subsequently used by conflict participants between 2011 and 2013).

Peaks in opposition political activity coincided with the Australian public consultation (April to June 2011, when JTI made 17 gifts to MPs), the UK public consultation (April to August 2012), the Department of Health announcement that they would ‘wait and see’ what evidence emerged from Australia (July 2013)^3^ ^140^ and the decision to establish the independent Chantler Review of evidence on standardised packaging (November 2013) (figure 4 and see online supplementary file 1).^26^ ^141^ Research publications peaked early in June 2012, midway through the consultation. Public communications dominated in the period leading into the consultation and again when the Chantler Review was announced. Direct lobbying occurred throughout but was particularly prevalent in the period after the consultation, when the government emphasised that it had an ‘open mind’.^142–144^

## Discussion

Nearly 9 of every 10 political activities undertaken to oppose standardised packaging were financially linked to the tobacco industry. Eighty-two diverse third party organisations with financial relationships with TTCs^58^ undertook 60% of all opposition activity, including the vast majority of research production (88%) and public communications (78%). Active organisations among them were rarely transparent about their relationship with TTCs. TTCs undertook 28% of activities themselves, including 64% of all direct lobbying. While previous research has identified TTCs' use of third parties,^54^ ^145^ this is the first paper to quantify the extent of TTC-supported political activity. It shows that the majority of activities undertaken to oppose standardised tobacco packaging in the UK were linked to the tobacco industry. The significance of this for public health policy debates should not be underestimated.

TTCs' long-term and campaign-specific support for third parties expanded the policy conflict^146^ ^147^ in two key respects. First, it increased the capacity of participants in the conflict to undertake opposition activities. Second, it helped mobilise the support of a diverse range of constituencies including the wholesale and retail sectors—which the official impact assessment found were likely to be only marginally affected by standardised packaging^46^—and general business and civil society groups—who had no direct financial interest in the outcome of the conflict. The high proportion of opposition organisations that received financial subsidies from TTCs highlights the power of industry money to induce and augment political action.^148–151^ Membership subscriptions and other long-term financial subsidies to organisations create a form of latent political capital that can be drawn on in the context of specific health policy conflicts. Industry funds research to underpin and arguably enhance the legitimacy of public communications and lobbying.^106^ ^152–154^ Direct campaign subsidies lower the costs of political activity, and increase the range of politically important constituencies that can be effectively targeted and mobilised by industry arguments.^155^ ^156^

These findings illustrate the importance of third parties to TTCs' *insider* and *outsider* political strategies^41^ ^42^ as they adapt to both Article 5.3 of the FCTC^35^ ^36^ and to Better Regulation.^37^ With regard to *insider* political strategies, TTC financial and in-kind support for third party policy-facing activities (direct lobbying, research production) is instrumental in off-setting government commitments under Article 5.3 to reduce tobacco industry access to policymakers. This is consistent with research which uncovered extensive third party industry lobbying against the European Union Tobacco Products Directive.^145^ ^157^ Subsidising research production also optimises TTCs' opportunities to capitalise on the importance of evidence in shaping mandatory impact assessments.^54^ ^55^

Subsidising public-facing third party activities (mass recruitment, public communication, research promotion) was central to the industry's *outsider* strategy. These activities aimed to expand the conflict by mobilising specific constituencies and the general public against standardised packaging at key points within the policy process. For example, TTC funding of mass recruitment campaigns during the 2012 consultation produced 98% of submissions objecting to the policy.^3^ The absence of an immediately observable link between these active third party activities and the industry is likely to have been instrumental in their effectiveness,^54^ helping to create an impression of strong opposition to the policy from a wider range of constituencies. This is consistent with efforts by active opponents of standardised packaging to present the consultation as a referendum on the policy,^158^ despite the wealth of supportive evidence,^18^ ^26^ the support of the majority of MPs^50^ ^159^ and opinion polls showing that only 11% of the general public opposed the measure.^160^ ^161^ This practice represents a highly public way of formally registering dissent to policy proposals^38^ ^145^ ^157^ ^162^ and presents further evidence of industry adaptation to the opportunities for influence inherent within Better Regulation processes.

The unique contribution of the study lies in the innovative combination of publicly available sources and investigative research techniques, which facilitates analysis of both the *scale* of political action in contemporary health policy conflicts and the degree of *support* provided by the tobacco industry. By making explicit the links between ostensibly independent organisations opposing tobacco policy and TTCs, the research aims to reduce the utility of their third party strategy for opposing public health policies. Further innovation comes from the systematic classification of the sector and relationships of actors engaged in political activity, which enables the examination of relationships between ostensibly distinct groups opposing public health policy. This is a novel system which could be applied to increase understanding of how corporations in other sectors responsible for producing commodities harmful to health (eg, alcohol, sugar-sweetened beverages) oppose population level policy instruments. By outlining the relationships between TTCs and other organisations in the conflict around standardised packaging and mapping these relationships to political actions, the study builds on the political science literature which primarily focuses on the role of civil society groups in expanding policy conflicts and understates the role of financial subsidies to conflict participants.^38^ ^57^ ^162–167^ The findings complement research examining tobacco industry arguments, strategies and tactics used in Australia to oppose standardised packaging.^168^

The ongoing nature of the policy process during the study period limited access to key sources of data. Officials from the Department of Health declined requests for interview. Some FOI requests were declined under section 35 of the Freedom of Information Act (FOIA), which exempts the release of information relating to ‘the formulation or development of government policy’^169^ ^170^ and the Regulatory Policy Committee (a key organisation responsible for approving impact assessments^40^) is not covered by FOIA.^171^ The practical requirements of data volume led to the exclusion of social media data from the study. It is thus possible that the volume of tobacco industry-supported activity and the extent and nature of relationships between TTCs and third parties were greater than is revealed by the study. Supplementary research using social network and social media analysis would provide deeper insights into how protobacco policy networks are formed and operate.

Our findings have major policy implications of relevance to parties to the FCTC and to countries where Better Regulation processes are integral to the policy process. First, and most importantly, the high incidence of financial relationships between tobacco manufacturers and third party opponents highlights the need for all policymakers, and the media,^172^ to treat organisations in tobacco policy conflicts with scepticism and to routinely require declarations of financial relationships in all interactions. To strengthen implementation of Article 5.3 of the FCTC, governance reforms should be introduced to require: (1) regular reports from TTCs on their affiliations, political activities and associated expenditure; and (2) the establishment of a transparent system of disclosure, through which all non-tobacco industry organisations lobbying any part of government on tobacco control must *always* disclose core funding, donations and membership fees from TTCs.

In addition, evidence of government-wide lobbying underlines the importance of health officials raising awareness and sharing information with other government departments regarding industry interference in public policy in accordance with the guidelines for implementation of Article 5.3.^35^ The scale and complexity of relationships between TTCs and other organisations engaged in tobacco policy conflicts reaffirms the importance of industry monitoring.^173^ ^174^ Finally, the timing and scale of industry-funded research, which, ultimately, feeds into stakeholder consultations and impact assessments,^44^ highlights the importance of continued funding of policy-relevant research by public and third sector organisations.
