# Correction: *Standardised tobacco packaging: a health policy case study of corporate conflict expansion and adaptation*

**DOI:** 10.1136/bmjopen-2016-012634corr1

**Published:** 2016-10-19

Hatchard JL, Fooks GJ, Gilmore AB. Standardised tobacco packaging: a health policy case study of corporate conflict expansion and adaptation. *BMJ Open* 2016;6:e012634. On page 8, a sentence reads: ‘For example, in public communications former police officers did not declare membership of the Common Sense Alliance (a FOREST offshoot).’ The Common Sense Alliance is not an offshoot of FOREST. It was supported by British American Tobacco among other organisations. The phrase in brackets ‘(a FOREST offshoot)’ should be omitted.

