# Supplementary material for: Standardised tobacco packaging: a health policy case study of corporate conflict expansion and adaptation
Source: BMJ Open. 2016 Oct 7;6(10):e012634. doi: 10.1136/bmjopen-2016-012634 (PMC5073631; doi:10.1136/bmjopen-2016-012634)
Supplement: supplementary data [file bmjopen-2016-012634supp.pdf]

Policy timeline: Standardised packaging of tobacco products in the UK 2008-2016	
2008	*May-September*, New Labour's *Consultation on the Future of Tobacco Control* tabled standardised packaging for the first time in the UK(1). Between 2008 and 2010, transnational tobacco companies commissioned 14 research reports on standardised packaging which were later used in the policy conflict.
2011	*March,* Conservative-Liberal Coalition Government committed to explore options for reducing the promotional impact of tobacco packaging in their *Tobacco Control Plan for England*(2).
2012	*February*, The Regulatory Policy Committee (a non-departmental body affiliated to the Department for Business, Innovation and Skills) gave the Department of Health's impact assessment on standardised packaging an amber rating(3).
	April-August, A four month UK-wide public consultation on standardised packaging was held by the Department of Health(4, 5). The consultation attracted 668,433 responses: 2,444 detailed responses of which 53% were supportive, 43% opposed; and 665,989 campaign responses (eg. postcards and standard letters or emails), of which 36% were supportive, 64% opposed. This represented the largest ever response to a public consultation in the UK and signalled a change in levels of public participation from 2008 when the more wide-ranging *Consultation on the Future of Tobacco Control*(1) attracted 96,515 responses.
	November, Public relations firm, Crosby Textor, were contracted by both Philip Morris International (PMI) and The UK Conservative Party. This later led to criticism of the Government as having been lobbied by Crosby Textor on standardised packaging. Prime Minister, David Cameron, denied he had been lobbied. However, documents released under Freedom of Information legislation revealed that Lynton Crosby of Crosby Textor did write to Intellectual Property Minister, Lord Marland, on behalf of PMI in November 2012 drawing attention to a legal opinion by Lord Hoffman commissioned by Philip Morris(6). In the same month, the four main transnational tobacco companies (British American Tobacco (BAT), Imperial Tobacco Group (ITG), Japan Tobacco International (JTI) and PMI) were sent invitations to attend meetings at the Department of Health.
2013	*January-February*, On invitation, representatives from all four major tobacco companies attended meetings with Department of Health officials to discuss the content of the impact assessment.
	May, Contrary to public and media speculation, standardised packaging was not in the Queen's Speech(4).
	July, The consultation report was published 11 months after the consultation closed. The Coalition Government announced a decision to 'wait and see' what evidence emerged from Australia, the only jurisdiction in the world to have implemented standardised packaging(7). In the same month, members of the House of Lords tabled an amendment to the Children and Families Bill which would pave the way for standardised packaging(8).
	September, Members of Parliament debated standardised packaging in the House of Commons(4).
	November, Under pressure from both Houses of Parliament, the Government commissioned an independent review of the evidence on standardised packaging led by Sir Cyril Chantler(9).
	December, The Government tabled their own amendment to the Children and Families Bill(10).
2014	*February*, the House of Commons supported an amendment to include enabling legislation for standardised packaging in the Children and Families Bill: 450 in favour, 21 opposed(4, 11).
	March, The *Chantler Review* of the evidence on standardised packaging supported implementation of standardised packaging, finding that '…the body of evidence shows that standardised packaging, in conjunction with the current tobacco control regime, is very likely to lead to a modest but important reduction over time on the uptake and prevalence of smoking and thus have a positive impact on public health'(12).
	June, A second impact assessment was published, again with an amber rating from the Regulatory Policy Committee(13), and a second public consultation was held on the text of the regulations(14).
	August, The views of all European Union (EU) Member States were invited under the EU's 98/34 procedure – comments were received from Ireland and Bulgaria, the Czech Republic, Greece, Hungary, Italy, Lithuania, Poland, Portugal, Romania, Slovakia and Spain submitted detailed opinions(15).
2015	*January*, The Coalition Government announced that a vote on standardised packaging would be held in the House of Commons before Parliament's dissolution in March for the 2015 general election.
	February, The Regulatory Policy Committee gave a third standardised packaging impact assessment a green rating, following significant revision responding to a change by the Regulatory Policy Committee in the definition of 'direct costs': "…policies which ban or severely restrict a particular activity, that explicitly prohibit a form of promotional activity, and have a primary objective to reduce sales (even if by promoting behaviour change) should be considered as having a direct impact on businesses. In this IA we therefore treat these profit losses as a direct impact for OITO purposes" (para 250, (16)). The impact assessment concluded that the overall benefit

	to the economy of standardised packaging would be £25bn, although transition costs to tobacco manufacturers, packagers, wholesalers and retailers from the introduction of standardised packaging were estimated at £400m (including reduced profits from loss of brand value).
	March, The House of Commons voted to introduce standardised packaging for tobacco products from May 2016, 367 in favour, 113 opposed(5, 17).
	May, PMI, JTI and BAT initiated legal action against the UK's standardised packaging legislation(18, 19). ITG followed suit.
	December, A 6 day court case took place at the UK's High Court – ruling 18th May 2016.
2016	*May,* The European Court of Justice upheld the legality of the standardised packaging clauses of the Tobacco Products Directive(20). The UK High Court ruled in favour of the UK Department of Health.(21) Standardised packaging was implemented in the UK on 20th May 2016.

REFERENCES

1. Department of Health. Consultation on the future of tobacco control London; 2008 [February 2016]. Available from: http://webarchive.nationalarchives.gov.uk/20130107105354/http://www.dh.gov.uk/prod_consum_dh/groups/dh_digitalassets/documents/digitalasset/dh_085651.pdf.
2. HM Government. Healthy Lives, Healthy People: A Tobacco Control Plan for England London; 2011 [February 2016]. Available from: https://www.gov.uk/government/uploads/system/uploads/attachment_data/file/213757/dh_124960.pdf.
3. Department of Health. Impact Assessment: Standardised packaging for tobacco products London: UK Government; 2012 [February 2016]. Available from: http://consultations.dh.gov.uk/tobacco/standardised-packaging-of-tobacco-products/supporting_documents/17194%20Impact%20assessment%20%20Standardised%20packaging%20of%20tobacco%20products%20final.pdf.
4. Barber S, Conway, L. Standardised (plain) packaging of tobacco products London: House of Commons Library; 2015 [updated 22.01.15February 2016]. Available from: http://www.parliament.uk/business/publications/research/briefing-papers/SN06175/standardised-packaging-of-tobacco-products.
5. Department of Health. Government backs standardised packaging of tobacco London; 2015 [updated 21.01.15February 2016]. Available from: https://www.gov.uk/government/news/government-backs-standardised-packaging-of-tobacco.
6. Crosby L. IP issues and plain packaging email to Lord Marland Parliamentary Under Secretary for Intellectual Property. Freedom of Information Request: Intellectual Property Office; 2012.
7. Department of Health. Consultation on standardised packaging of tobacco products: Summary report London; 2013, July [February 2016]. Available from: https://www.gov.uk/government/consultations/standardised-packaging-of-tobacco-products.
8. Lord Faulkner Baroness Tyler BF, Lord McColl,. Children and Families Bill: Amendments to be moved in Grand Committee - Children's health: Standardised tobacco packaging, 20th July 2013. In: House of Lords, editor. 2013.
9. Ellison J. Written Ministerial Statement: Department of Health - Tobacco control update UK: Hansard; 2013 [updated 28th November 2013February 2016]. Available from: http://www.parliament.uk/documents/commons-vote-office/November-2013/28%20November/7-Health-TobaccoControl.pdf.
10. UK Government. Amendment to the Children and Families Bill. In: Department of Health, editor. Hansard, UK 2013.
11. UK Government. Children and Families Act 2014 Chapter 6 London: UK Government; 2014 [February 2016]. Available from: http://www.legislation.gov.uk/ukpga/2014/6/contents/enacted.
12. Chantler C. Standardised packaging of tobacco: Report of the independent review undertaken by Sir Cyril Chantler London: King's College London; 2014 [February 2016]. Available from: http://www.kcl.ac.uk/health/10035-TSO-2901853-Chantler-Review-ACCESSIBLE.PDF.
13. Department of Health. Impact Assessment: Standardised packaging of tobacco products London: UK Government; 2014 [February 2016]. Available from: https://www.gov.uk/government/uploads/system/uploads/attachment_data/file/323518/impact_assessment.pdf.
14. Department of Health. Consultation on the introduction of regulations for standardised packaging of tobacco products. London: Williams Lea; 2014.
15. Department of Health. 98/34 Notification: The standardised packaging of tobacco product regulations. Brussels, Belgium: European Commission; 2014.
16. Department of Health. Standardised packaging of tobacco products: Impact Assessment London: UK Government; 2015 [updated 10.02.15February 2016]. Available from: https://www.gov.uk/government/uploads/system/uploads/attachment_data/file/403493/Impact_assessment.pdf.
17. UK Government. Public health: The standardised packaging of tobacco products regulations UK: The Stationery Office Ltd.; 2015 [February 2016]. Available from: http://www.legislation.gov.uk/ukdsi/2015/9780111129876/pdfs/ukdsi_9780111129876_en.pdf.
18. Cooper C. Tobacco companies file lawsuits against UK Government over plain packagign laws. The Independent. 2015 22nd May 2015. Available from: http://www.independent.co.uk/life-style/health-and-families/health-news/tobacco-companies-file-lawsuits-against-uk-government-over-plain-packaging-laws-10270874.html.
19. Corbin T. Anti-plain pack legal action grows as JTI files suit. Packaging News. 2015 27th May 2015. Available from: http://www.packagingnews.co.uk/news/markets/tobacco/anti-plain-pack-legal-action-grows-jti-file-suit/.
20. European Court of Justice. PMI, BAT vs Department of Health UK Luxembourg; 2016. Available from: http://curia.europa.eu/juris/document/document.jsf?text=tobacco&docid=177724&pageIndex=0&doclang=EN&mode=req&dir=&occ=first&part=1&cid=391272#ctx1.
21. Royal High Courts of Justice. Tobacco companies vs. UK Department of Health London, UK; 2016. Available from: https://www.judiciary.gov.uk/wp-content/uploads/2016/05/bat-v-doh.judgment.pdf.
